# Current Trends in Biohumoral Screening for the Risk of Sudden Cardiac Death: A Systematic Review

**DOI:** 10.3390/medicina60030418

**Published:** 2024-02-29

**Authors:** Oana-Maria Isailă, Lavinia-Alexandra Moroianu, Sorin Hostiuc

**Affiliations:** 1 Department of Legal Medicine and Bioethics, Faculty of Dentistry, “Carol Davila” University of Medicine and Pharmacy, 020021 Bucharest, Romania; 2 Clinical Medical Department, Faculty of Medicine and Pharmacy, Dunarea de Jos University, 47 Domneasca Street, 800008 Galati, Romania; lavinia.moroianu@yahoo.com

**Keywords:** screening, biomarkers, sudden cardiac death, specificity

## Abstract

*Background and Objectives*: Sudden cardiac death (SCD) represents a challenge to health systems globally and is met with increased frequency in the population. Over time, multiple screening methods have been proposed, including the analysis of various plasma biomarkers. This article aims to analyze for illustrative purposes the specialized literature in terms of current biomarkers and testing trends, in the case of cardiovascular diseases and implicitly sudden cardiac death. *Materials and Methods*: In this regard, we searched the PubMed database from 2010 to the present time using the keywords “sudden cardiac death” and “biomarkers”. The inclusion criteria were clinical trials that analyzed the effectiveness of screening methods in terms of biomarkers used in stratifying the risk of cardiac distress and/or sudden cardiac death. We excluded reviews, meta-analyses, and studies looking at the effectiveness of treatments. *Results*: An extended approach was found, through studies that brought to the forefront both classical markers analyzed by new, more performant methods, markers for other pathologies that also determined cardiovascular impact, non-specific molecules with effects on the cardiovascular system, and state-of-the-art markers, such as microRNA. Some molecules were analyzed simultaneously in certain groups of patients. *Conclusion*: The observed current trend revealed the tendency to define the clinical-biological particularities of the person to be screened.

## 1. Introduction

Sudden cardiac death (SCD) is the unexpected death within an hour of symptom onset or during sleep in a healthy person with no documented personal pathological history [1]. It accounts for 10–15% of deaths globally [2] and is a challenge for health systems. Its annual incidence is constantly increasing and is more common in men [3]. Traditional population factors associated with sudden cardiac death are considered: male gender, race, old age, low socioeconomic status, obesity, and metabolic disorders [4]. Causative factors in sudden cardiac death include arrhythmogenic genetic pathologies, cardiomyopathies, myocarditis, coronary pathology, and valvulopathies [5,6,7]. Sudden cardiac death has become a worrying phenomenon among young people lately, with a meta-analysis in this area finding among young athletes a prevalence of ischemic pathology, dilated cardiopathy, hypertrophic cardiomyopathy, and coronary abnormalities, and among young non-athletes, coronary artery disease, arrhythmogenic cardiomyopathy, channelopathies, and aortic dissection. Regarding the geographical region, in the same meta-analysis, it was observed that the prevalence of hypertrophic cardiomyopathy and coronary abnormalities was higher in the USA, while channelopathies prevailed in Europe [8]. Other authors correlate sudden cardiac death with family history, low educational attainment, non-sanogenic behaviors, including smoking, and non-regular physical activity—elements that showed variations according to the person’s age and sex [9]. In addition, gender, psychological, personal, community, and professional factors were considered [10]. For example, the National Heart Foundation in a survey found that only 55% of men and 39% of women over 45 years old in Australia had undergone a cardiological evaluation in the past two years [11]. Although potential effective screening methods are being explored, the phenomenon of sudden cardiovascular death still has a significant incidence. Screening methods can be electrophysiological (ECG)—in a recent meta-analysis the higher accuracy of ECG was compared to clinical examination in predicting sudden cardiac death in young athletes [12]—and blood pressure measurement, noting that high systolic pressure is associated with sudden cardiovascular death [13], and analysis of genetic markers as well as biohumoral markers [14,15]. The risk of sudden cardiovascular death can be stratified according to the following criteria (Table 1):

The purpose of this review is to analyze current biomarkers and testing trends, for illustrative and non-limiting purposes, for the assessment of sudden cardiac death risk.

## 2. Materials and Methods

We conducted a study in adherence to the PRISMA guidelines for reporting systematic literature reviews [17]. In this regard, we searched the PubMed database from 2010 until the present time using the keywords “sudden cardiac death” and “biomarkers”. The inclusion criteria were clinical trials that analyzed the effectiveness of screening methods in terms of biomarkers used in stratifying the risk of cardiac distress and/or sudden cardiac death. We excluded reviews, meta-analyses, and studies examining the effectiveness of treatments.

## 3. Results

**Figure 1:**
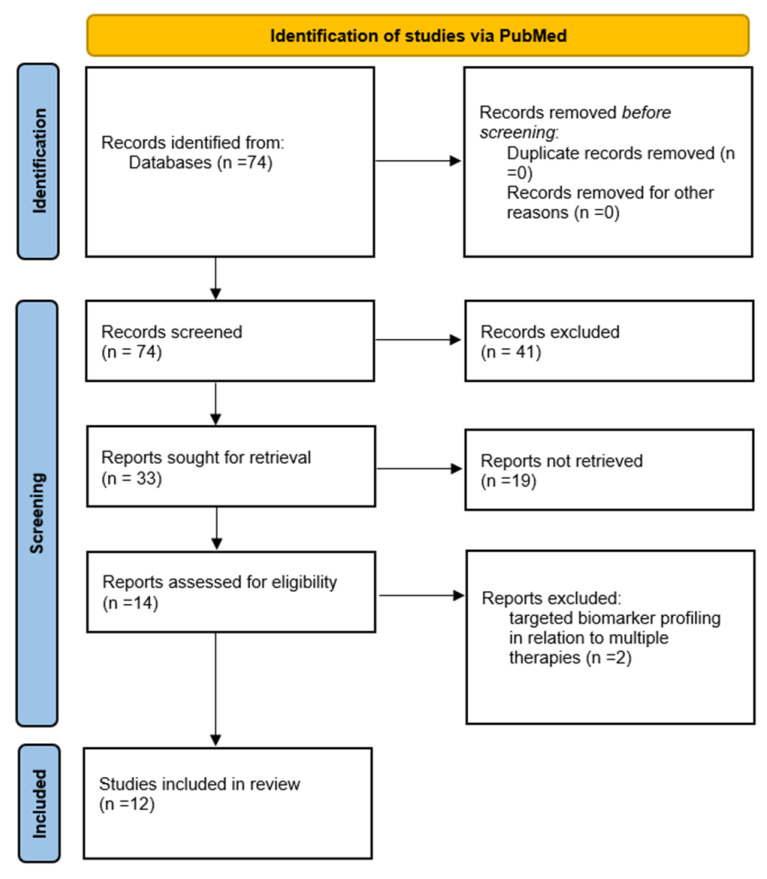
Search synthesis. Prisma flow diagram [17].

Seventy-four studies resulted. Following their analysis based on the inclusion criteria, we have included 12 articles for consideration in this review, as follows (Figure 1, Table 2, Table 3 and Table 4):

### 3.1. SCD Potential Biomarkers

#### 3.1.1. Homoarginine

Homoarginine is a nonproteinogenic amino acid homologous to L-arginine that can come from foods such as tomatoes, peas, lentils and is almost completely absorbed in the jejunum and ileum [30,31] but also occurs endogenously enzymatically in the kidney, liver, and pancreas, with exogenous intake playing a minor role [31]. L-arginine is the substrate from which Nitirc Oxide (NO) is synthesized under the action of the enzyme NO endothelial synthase. NO plays a role in regulating vascular tone, vascular resistance, arterial stiffness, and leukocyte function [32]. Circulating homoarginine concentrations were found to be in an inverse relationship with markers for cardiovascular dysfunction [33,34,35].

The Dallas Heart Study showed that there is a link between low homoarginine levels and thickening of the aortic wall. However, no such association was found in relation to arterial calcification. The study concluded that homoarginine is inversely associated with subclinical vascular pathology and may reduce the risk of developing cardiovascular complications [36]. A recent study investigating the association between homoarginine levels and atrial fibrillation or complications following a stroke resulted in positive associations between elevated homoarginine levels and a low number of complications, including a lower prevalence of atrial fibrillation and reduced carotid-intima media thickness [37]. On the other hand, another study found that homoarginine is associated with hyperglycemia and abdominal obesity in men, and type 2 diabetes in both sexes, without detecting a causal association in this regard, concluding that exposure to elevated circulating homoarginine levels does not seem to alter the risk of cardiometabolic disease [38]. Ali et al. conducted a study on 7638 individuals and found homoarginine to be a potential marker of liver dysfunction when correlated with common liver biomarkers [39]. A meta-analysis of observational studies examining the relationship between homoarginine and all-cause mortality discovered a significant association between low homoarginine levels and death [40].

#### 3.1.2. Serum 25-hydroxyvitamin D

Serum 25-hydroxyvitamin D, a circulating biomarker of vitamin D, one of the most important steroid hormones, is synthesized in the liver by 25-hydroxylation from inactive precursors [41]. It is fat-soluble and plays an important role in mineral homeostasis. In addition to its effects on the skeletal system, it is also an important factor in other tissues, vitamin D methanolites being involved in skin diseases, autoimmune diseases, neoplasias, diabetes, and cardiovascular pathologies [42]. The association between vitamin D and cardiovascular disease has been assumed in the past with the observation of increased cardiovascular events in winter [43]. It was found that it plays a role in the cardiovascular system by modulating inflammation and immune response, keeping them within normal limits [44], decreasing the risk of thrombosis [45], and decreasing cardiac remodeling through antihypetrophic effects [46]. The association between 25-hydroxyvitamin D levels and vascular calcification is questionable, with studies showing divergent results [47,48,49].

A recent 2023 meta-analysis of prospective studies on this topic noted a positive association between low circulating vitamin D levels and significantly increased risk of sudden cardiac death and cardiovascular events [50]—results in agreement with another similar meta-analysis conducted in 2017 [51]. Gholami et al., also in a meta-analysis of prospective-cohort studies in which they analyzed this association, also found the protective role of serum 25-hydroxyvitamin D in the cardiovascular system, noting the need for extensive evaluation also according to the sex of patients [52]. Regarding specificity, for example, purposes, we mention that positive associations were also found between the negative prognosis of primary membranous nephropathy and low levels of serum 25-hydroxyvitamin D, being considered a potential marker in this regard as well [53]. Also, 25-hydroxyvitamin D is considered a potential biomarker of sickle cell disease, given its positive correlation with hemolysis markers [54].

#### 3.1.3. Thyroid-Stimulating Hormone (TSH)

Thyroid hormones are known for their repercussions on cardiovascular function, with effects on heart rate, cardiac contractility, and vascular resistance, even when thyroid dysfunction is subclinical, with changes only in TSH and T3, T4 values within normal limits [55,56]. Numerous studies have analyzed the correlation between subclinical hypothyroidism and the carotid intima-media thickness observed by ultrasound as a cardiovascular risk factor, establishing a positive association [57,58,59] and proving the atherosclerotic role of subclinical hypothyroidism. Chaker et al. studied the correlation between thyroid function and sudden cardiac death and suggested an increased risk of sudden cardiac death in cases with elevated FT4 values [60]. Meta-analysis by Sun et al. in 2017 described with reservations a positive association between subclinical hypothyroidism and increased risk of coronary pathology, cardiac death, and mortality in general [61]. However, there are not enough studies to demonstrate the direct tanatogenerative effects of cardiovascular pathology following thyroid dysfunction, and implicitly the risk of sudden cardiovascular death in these cases. Its direct use for assessing the risk of sudden cardiac death is questionable, there being no elements of sensitivity or specificity in this regard.

#### 3.1.4. NT-pro BNP

The three important natriuretic peptides (NP) are ANP (atrial), BNP (brain) and CNP (type C). Normally, the production of ANP and BNP occurs in the atria. However, in some cases of illness, BNP production may also occur in the ventricles. This can result in a significant increase in BNP levels in the bloodstream, up to 5–10 times higher than ANP levels [62]. At the intracellular level, BNP activates molecules such as cGMP-dependent protein kinase and phosphodiesterase. These molecules play a role in ion channel closure, salt and water excretion, vasodilation, and have anti-inflammatory and antiapoptotic properties [63]. N-terminal pro-B-type natriuretic peptide (NT-pro BNP) is an inactive terminal molecule released in proportions equal to BNP but with greater plasma stability and a longer half-life (90–120 min), which determines its preferential use as a biohumoral marker in cardiac pathologies [62]. The dosage of NT-pro BNP is considered useful in terms of additional contribution to the prediction of cardiovascular events, including among individuals without a history of cardiovascular disease, as demonstrated in a meta-analysis of 40 prospective studies in 12 countries [64]. Meta-analyses have shown the predictive role of NT-pro BNP in cardiovascular events among patients with chronic cardiovascular diseases, such as chronic heart failure [65,66]. At the same time, the meta-analysis conducted by Cao et al. on necropsy studies exposes higher levels of NT-pro BNP in pericardial fluid in cases of sudden cardiac death compared to those of sudden non-cardiac death, suggesting postmortem NT-proBNP from pericardial fluid may be considered as an indicator in assessing agonal heart function [67]. However, BNP/NT-proBNP values can also be influenced by other factors such as kidney function disorders, aortic stenosis, and pulmonary hypertension, which give increased results [68], while obesity can cause low values in this test [69], which makes the accuracy of the test low [70]. In a necropsy study, NT-proBNP dosing showed a specificity of 72.6% and a sensitivity of 50.7% for heart failure [71].

#### 3.1.5. Galectin 3

Galectin 3 belongs to the family of beta-galactoside-binding proteins. So far, 17 types of galectins have been identified, but galectin 3 has been the most studied of these, being found both intracellularly and extracellularly in various tissues [72]. Galectin 3 is associated with inflammatory processes and myocardial fibrosis, being found to be present at the myocardial level after the onset of ischemia [73]. There are studies that have correlated increased levels of galectin 3 with increased mortality, including cardiovascular mortality, being considered an independent predictor in this regard [74,75]. A 2015 study found that elevated galectin 3 levels in hospitalized patients with heart failure confer an additional negative prognosis, but without predictive power of death [76]. A recent meta-analysis also observed a statistically significant association between elevated galectin 3 levels and risk of heart failure [77]. A similar meta-analysis from 2020 suggested a positive association between elevated galectin 3 levels and all-cause mortality among people who had suffered an acute myocardial infarction [78].

Elevated levels of galectin 3 can also be found in other pathologies such as neoplasms (e.g., malignant thyroid neoplasms [79]) and other fibrotic or inflammatory pathologies [80]. According to some authors, it is considered a marker of thyroid cancer [81].

#### 3.1.6. ST2

ST2 belongs to the group of receptors for interleukin 1 (IL1), a marker with cardiac tropism [82]. The soluble circulating form, ST2, is thought to reflect cardiac stress and is also a predictor of cardiovascular disease among people without a personal pathological history, with potential utility in stratifying cardiovascular risk [83]. There are studies that postulate that ST2 would play a role in the pathophysiology of myocardial fibrosis [84] but also in the prognosis of morbidity and mortality after acute myocardial infarction [85]. On the other hand, a case-control study advocates a more reserved use of ST2 dosing, or interpretation of ST2 dosing results in combination with other cardiac markers, following 3T cardiac magnetic resonance analysis, finding that ST2 does not help detect myocardial fibrosis or myocardial inflammation [86]. A study analyzed ST2 levels in patients with heart disease, acute myocardial infarction, and heart failure. The results concluded that high ST2 levels were significantly associated with heart failure alone, with no significant differences in ST2 levels found between healthy individuals and patients with ischemic heart disease or acute myocardial infarction. The authors recommended using ST2 as an additional marker in the diagnosis of heart failure [87]. Elevated levels of ST2 as a negative prognostic factor can also be found in myelodysplastic syndrome [88], pulmonary hypertension [89], ulcerative colitis [90], and idiopathic pulmonary fibrosis [91].

#### 3.1.7. Fasting Plasma Leptin

Leptin is a hormone produced in adipose tissue, correlated with obesity and considered a link between obesity and cardiovascular pathology [92]. It plays a role in regulating food intake and satiety [93] as well as regulating blood pressure, activation of the sympathetic nervous system, insulin resistance, arterial thrombosis, angiogenesis, and vascular inflammatory response [94,95]. In vitro, studies have found that leptin increases vascular endothelial growth factor (VEGF) synthesis, a marker of endothelial dysfunction [96,97]. In a prospective study of 361 patients, the role of leptin as an independent predictor of cardiovascular events in patients with angiographically confirmed coronary atherosclerosis was postulated, without being influenced by the lipid profile or C-reactive protein (CRP) [98]. A similar recent study of 971 patients found similar results [99]. A meta-analysis of the correlation between elevated leptin levels and coronary pathology found no statistically significant association overall in women and men, suggesting higher risks among males [100].

Jacobsson et al., in a study of 152 patients, correlated hyperleptinemia with sepsis and poor prognosis of sepsis in men [101]. A meta-analysis by Zhu et al. suggested a positive association between elevated leptin levels and periodontitis [102].

#### 3.1.8. 9-cis-Retinoic Acid (9 cRA)

Metabolomics involves research into modifications of finished products in vivo following the application of certain stimuli within a biological system [103]. This allows for a more comprehensive, real-time understanding of the disease course [104]. In this regard, 9-cis-retinoic acid (9 cRA), an active metabolite of vitamin A, has also begun to be analyzed as a potential marker within cardiovascular pathology. For example, the PRIME study found a decrease in retinol levels in middle-aged men without clinical signs but at increased risk of cardiovascular events [105]. Luo et al., in a case-control study, subject to study reproducibility, found that retinol metabolism was a detectable discriminative pathway in early detection of ventricular fibrillation in post-STEMI patients [106]. Huang et al., in a study preceding the aforementioned one, presented retinol metabolism as the most significant differential pathway in the pathogenesis of left main coronary artery disease, with STEMI previously stating that 9-cis-retinoic acid had the highest discriminating value [24]. However, the number of studies investigating this correlation is small, and 9-cis-retinoic acid levels have been analyzed and positively correlated with inflammatory pathologies [107,108].

#### 3.1.9. High-Sensitivity Troponin (Hs-TnT)

Troponins are structural proteins found in both skeletal muscle and heart muscle. Troponin T is one of the troponins considered exclusively cardiac and considered a classical, specific biomarker of myocardial distress [109]. Being one of the usual markers of acute myocardial infarction, its values are analyzed in usual medical practice by immunochemical methods [110]. In order to measure Troponin T in recent years, high-sensitive methods have also appeared [111]. Much lower concentrations of this protein can be detected with greater precision than classical methods, providing early identification of acute myocardial infarction. On the other hand, through this test method, variations in TnT levels were found depending on the time of day, sex, and comorbidities, which can be erroneously interpreted as myocardial damage [112]. A meta-analysis of the correlation between Hs-TnT and heart failure, including 67.063 patients from prospective studies, found a strong positive association between Hs-TnT levels and the prediction of heart failure [113]. A recent meta-analysis, which included 11 studies examining the levels of Hs-TnT and cardiovascular risk, concluded that it has a strong predictive value for both all-cause and cardiovascular mortality risk in the general population [114]. Hs-TnT can also be analyzed postmortem from a pericardial fluid sample, making an essential contribution in the diagnosis of sudden cardiac death, without the installation of specific macroscopic changes [115].

#### 3.1.10. High-Sensitivity C-Reactive Protein (HS-CRP)

C-reactive protein (CRP) is a protein nonspecific inflammatory marker of the acute phase, first discovered as synthesized in the liver and released into the blood [116]. With the evolution of technologies for detecting this protein, the name high-sensitivity C-reactive protein appeared, which refers to conventional CRP detected by a high-sensitivity test, a new modified assay that allows the identification of plasma CRP even at very low levels, conferring the inflammatory status of the body [117]. It is currently considered to have predictive value of the risk of cardiovascular events, stroke, and peripheral arterial disease among people without clinical manifestations and medical history in this regard [118]. In a study analyzing the risks involved in the presence of high-sensitivity C-reactive protein among patients with hypertrophic cardiomyopathy, it was found that at values above > 3.0 mg/L (high hsCRP level), there were higher risks of cardiovascular death, all-cause mortality, and SCD, and concluded that hsCRP is an independent predictor of the aforementioned risks [119]. A similar recent study of 86.234 participants without cardiac conduction disorders revealed that elevated hsCRP levels are an independent risk factor for cardiac conduction disorders [120]. The meta-analysis of the predictive role of CRP, which included 12 prospective studies with a total of 36.646 persons, found CRP to be an independent predictor of sudden death [121]. Another recent meta-analysis during 2023 of patients with coronary artery disease undergoing percutaneous coronary intervention found a positive correlation between increased baseline hsCRP levels and increased risk for major adverse cardiac and cerebrovascular events, restenosis, and death [122].

However, CRP is a non-specific inflammatory marker and may present elevated blood values in inflammatory processes of various etiologies (bacterial, autoimmune, neoplastic, metabolic) [123]. Li et al., in a meta-analysis of this topic, concluded that elevated hsCRP levels may stratify the risk of cardiovascular death in the general population, being an independent predictor without making the same assessments about the risk of death from neoplastic causes [124].

#### 3.1.11. Albumin

Albumin is a protein synthesized in the liver, detectable in plasma, with multiple important physiological roles, such as maintaining plasma oncotic pressure, and antioxidant, anti-inflammatory, and anticoagulant properties [125]. From the point of view of its impact at the cardiovascular level, it was found that in patients with acute coronary syndrome and hypoalbuminemia (<3.5 g/dl), adverse results occurred more frequently during hospitalization, hypoalbuminemia being associated with a 2.8 times higher risk of finding complications such as reinfarction, acute heart failure, and cardiogenic shock, in their case [126]. In addition, in patients with heart failure, hypoalbuminemia was a marker of mortality [127,128]. In a recent prospective study of 1070 patients with cardiovascular pathology, an association was found between low albumin levels and long-term mortality in cardiovascular disease [129]. Another study of 2414 patients admitted to the ICU concluded that the higher the albumin levels at the time of intake, the lower the mortality rate was in patients with cardiac arrest [130]. In a meta-analysis published in 2022, which looked at the association between serum albumin levels and cardiac arrest outcomes in 3837 patients from observational studies, serum albumin levels measured in the early post-cardiac arrest phase were found to be significantly higher among surviving patients [131]. However, hypoalbuminemia has been studied and considered a negative prognostic factor in other pathologies such as acute kidney injury [132,133] or COVID-19 infection [134].

#### 3.1.12. Plasma Omega 3 Fatty Acids

The Omega 3 index was defined in 2004, and a highly standardized analytical laboratory methodology is necessary for its determination. It is considered a potential biomarker of cardiovascular events, including sudden cardiovascular death [135,136]. The interconnection of omega 3 fatty acids and sudden cardiac death started from studies that found the cardioprotective role of omega 3 administration through their antiarrhythmic effect [137]. However, some studies have not reached this conclusion because they have not observed a reduction in the incidence of sudden cardiac death following dietary supplementation with omega 3 fatty acids [138]. There were also no statistically significant results in a recent similar meta-analysis [139]. Regarding plasma omega 3 fatty acids and the risk of sudden cardiovascular death, the MERLIN-TIMI 36 study concluded that in patients with N-STEMI, plasma levels of omega 3 fatty acids are inversely associated with the risk of sudden cardiac death, independent of traditional risk factors [28]. The meta-analysis by Harris et al. concluded that increased plasma levels of omega 3 fatty acids were associated with decreased risk of all-cause mortality [140].

#### 3.1.13. MicroRNA-208-3p, microRNA-143-3p

In general, death in young people and under conditions of the non-conclusive autopsy, functional and electrophysiological causes of death should be investigated, which usually include cardiac arrhythmias of genetic causes. This type of investigation involves genetic analysis to detect possible genetic mutations [141]. In this regard, microRNAs or miRNAs have been elements of interest regarding their potential as biomarkers in various cardiovascular diseases [142]. MicroRNAs are endogenous noncoding RNAs of small size (~22 nucleotides) that influence biological processes, being regulators of cardiovascular function [142,143,144,145]. For example, microRNA-208 is considered myocardial tissue-specific [146] and essential in maintaining myocardial conduction and a potential marker in acute myocardial infarction (MI) [147]. In a meta-analysis that analyzed the miRNA profile for heart failure, they found miR-21, miR-30c, miR-210-3p, let-7i-5p, miR-129, let-7e-5p, and miR-622 as potential biomarkers in this regard [148]. In the study conducted by Pinchi et al. on necropsy casuistry, it was found that micro-RNA 208 (miR-208) showed the highest specificity in diagnostic differentiation of IMA [149]. In another study of miR-208a post STEAMI levels, relative serum levels of miR-208a were found to be at least 215 times higher in STEAMI patients than in healthy individuals [150]. Regarding microRNA-143-3p, its association with acute myocardial infarction and myocardial fibrosis was found in necropsy samples [151]. Tie et al., in a recent necropsy casework study, did not observe a conclusive association between miR-143 and the risk of coronary artery disease [152], whereas Satake et al., in a follow-up MI study, concluded that miR-143 in elevated plasma concentrations in the acute phase of MI may be a predictor of left ventricular function recovery in the chronic phase [153]. Other studies that have analyzed the role of micro-RNA-143-3p have found that it has anti-oncogenic activity in metastatic proliferation from colorectal cancer [154], suppresses tumor proliferation in pancreatic ductal adenocarcinoma [155], suppresses cell growth and invasion in laryngeal squamous cell carcinoma [156], attenuates the development of liver fibrosis in autoimmune hepatitis [157], and can also be considered a biomarker of gastric cancer [158].

## 4. Discussion

Theoretically, the ideal biomarker for sudden cardiac death involves meeting the following criteria: to be affordable and practical, to involve minimal costs, to have low biological variability, low variability of the analysis, to be evaluated in clinical trials, to have additive value, sensitivity, specificity, a good predictive value, to have utility in clinical decision making, and to have adequate calibration and discrimination [159].

Analyzing the trends in the search for potential biomarkers of sudden cardiac death from 2010 until the present time, the studies included in this review focus on new types of markers, as well as new techniques for detecting classical markers, or a new perspective on markers specific to other non-cardiovascular pathologies. In addition, the analysis of these markers is found frequently in clinical studies conducted among persons with clinically manifest, documented cardiovascular pathologies, which were an indicator of testing.

From the point of view of biological sampling for testing, small amounts of blood are required for all the above-mentioned biomarkers in the living person, which implies an easy, reproducible method with minimal invasiveness. In terms of dosing these markers, most laboratory techniques involve assay technology [160] or chromatography [161]. For miRNA techniques, next-generation sequencing, microarrays, and quantitative reverse transcription PCR are currently the most widely used [162].

For most of the markers analyzed, the aspects related to their sensitivity and specificity for cardiovascular pathology were not specified; some studies even analyzed the associated efficiency of several different markers simultaneously. It can be stated that the real tendency was to accurately seek the association of biohumoral factors to detect in a timely manner the negative prognosis of cardiovascular disease depending on the patient, and not the ideal biomarker, which opened multiple avenues of analysis such as metabolomics, proteomics, and genetics.

### 4.1. Ethical Aspects of Screening Tests for Cardiovascular Disease

Screening involves identifying an asymptomatic pathology prior to its clinical manifestation or identification of risk factors. The purpose of screening is to detect a disease in asymptomatic individuals and prevent its progression through secondary prevention. A crucial factor in recommending screening is the availability of effective therapies that can halt the progression of the disease, especially when screening methods with high sensitivity and specificity are employed. Screening must also be differentiated from risk estimation, risk estimation assuming the absence of disease at the time of testing and identification of the likelihood of future events related to the disease under consideration [163]. In the case of screening methods for pathologies with drastic prognosis, such as genetic diseases that can cause sudden cardiac death, appropriate pre- and post-testing medical counseling is required due to the anxiety generated for the patient and the family [164]. The issues of possible false positives, financial implications, and accessibility of screening resources that condition accessibility and recommendation of screening [165,166] should also be considered as a corollary.

### 4.2. Applicability of the Study

Although it has been shown for a long time that periodic ECG assessment and cardiac ultrasound, together with the appropriate assessment of the patient’s clinical condition, his main diagnoses and comorbidities, against which life-threatening rhythm disorders may develop, taking into account the patient’s family history, can allow timely detection of warning signs and effective prevention of SCD [12], in some cases they do not provide relevant information, and in cases of postmortem necropsy, biohumoral investigations are the only possible way to detect cardiac death in the absence of histopathological changes or medical history in this regard.

Also, with the evolution of molecular genetic methods, a large proportion of sudden cardiac deaths are attributed to genetic mutations (for example, genes that determine the development of channelopathies, cardiomyopathies, and other diseases, against the background of which life-threatening rhythm disorders can occur) [13,14] and subject to possible false-positive results. Currently, accessibility to genetic testing differs depending on the case and country, and involves certain ethical considerations such as discriminatory use of genetic data and the risk of genetic discrimination related to limited employability or insurability, lowering the person’s socio-economic status, which also is a risk factor for sudden cardiac death [167]. Therefore, until there is complete regulation of the new, effective genetic screening, in the best interest of the persons, the classic biohumoral testing methods still represent a screening option.

One potential current application of biohumoral testing for sudden cardiac death is the development of rapid test kits [168], which, due to their ease of use, would increase accessibility of screening in the absence of other methods available in some cases.

### 4.3. Limits of the Study

This study did not analyze all databases, and therefore did not cover the entire plethora of biomarkers recently exposed in the literature. In the analyzed database, we did not find translational studies that validate biohumoral testing in this regard, only clinical trials.

Also, another limitation of this review is represented by the absence of studies focused on channelopathies, cardiomyopathies, as well as the usual risk factors, such as age and gender.

However, overall, the recent trend in sudden cardiac death screening is accurately reflected in terms of the main techniques and biomarker classes.

## 5. Conclusions

Cardiovascular pathology and cardiovascular death, including sudden cardiac death, remain issues of major concern for health systems. There is still a need for biohumoral screening methods that allow adequate patient management to avoid an unfavorable prognosis, a trend that may currently involve genetic testing techniques. This screening must take into account the physiological and pathological characteristics of the person, including the assessment of personal and family history. In biohumoral screening, the person who should be screened prevails, considering the medical and, from case to case, socio-professional measures imposed following a positive screening.

## Figures and Tables

**Table 1:** Risk assessment of sudden cardiovascular death according to Osman et al., 2019 [16].

Assessment of risk factors	Age Sex Smoking Hypertension Obesity Hypercholesterolemia Diabetes Family history
Electrophysiological evaluations	EKG
Ultrasound evaluations	Left ventricular ejection fraction (LVEF)
Laboratory tests	Genetic biomarkers Protein biomarkers Other molecular biomarkers

**Table 2:** Potential biomarkers analyzed from 2010 to the present.

First Author Year	Biomarker	Observations
Drechsler et al., 2011 [18]	Homoarginine	Screening in hemodialysis patients. Low homoarginine levels were a risk factor for SCD and death due to heart failure.
Naesgaard et al., 2012 [19]	Serum 25-hydroxyvitamin D	Screening in chest pain patients. Useful biomarker for mortality prediction in patients with suspected acute coronary syndrome associated with elevated TnT levels.
Perez et al., 2014 [20]	Thyroid-stimulating hormone (TSH) NT-proBNP	Screening for sudden cardiac death in heart failure. No association between hypothyroidism and all outcomes.
Ahmad et al., 2014 [21]	NT-proBNP, galectin-3, and ST2	Screening of patients with chronic heart failure. Clinical predictors along with NT-proBNP levels were strong predictors of pump failure risk, with insignificant incremental contributions of ST2 and galectin-3.
Skali et al., 2016 [22]	Soluble ST2	Screening of patients with mildly symptomatic heart failure. Elevated baseline sST2 levels were associated with an increased risk of cardiovascular death.
Puurunen et al., 2017 [23]	Fasting plasma leptin	Screening of coronary artery disease patients. High plasma leptin levels predict the short-term occurrence of congestive heart failure or cardiac death and acute coronary syndrome or stroke independently of established risk factors.
Hunag et al., 2018 [24]	9-cis-retinoic acid (9cRA)	Screening of STEMI patients. 9cRA was the most critical biomarker of left main coronary artery disease.
Sharma et al., 2018 [25]	High-sensitivity troponin T, growth differentiation factor-15, NT-proBNP	Screening of patients with atrial fibrillation. Doubling of troponin T was strongly associated with sudden cardiac death, NT-proBNP with heart failure.
Everett et al., 2020 [26]	Total to high-density lipoprotein cholesterol ratio, high-sensitivity cardiac troponin I, NT-proBNP (N-terminal pro-B-type natriuretic peptide), or high-sensitivity C-reactive protein individually or in combination	Screening in the low-risk population. These measures may have utility in identifying individuals at risk of SCD.
Rhode et al., 2021 [27]	Albumin Uric acid Total bilirubin	Screening of chronic heart failure patients. Blood urea nitrogen and albumin levels were associated with a differential risk of sudden vs. non-sudden cardiovascular deaths.
Zelniker et al., 2021 [28]	Plasma Omega-3 Fatty Acids	Screening in patients after an acute coronary syndrome. In patients after non-ST-segment-elevation-acute coronary syndrome, plasma long-chain ω3-PUFAs are inversely associated with lower odds of sudden cardiac death, independent of traditional risk factors and lipids.
Huang et al., 2023 [29]	MicroRNA-208b-3p MicroRNA-143-3p	Screening of acute coronary syndrome patients. Positive predictive power of cardiovascular death confirmed in both the living person and postmortem.

**Table 3:** Current trends in search for potential biomarkers related to the patient.

Medical Particularities of the Persons Analyzed	Number of Studies
Chronic heart failure patients	4 studies
Acute coronary syndrome patients	4 studies
Chest pain patients	1 study
Atrial fibrillation patients	1 study
Low risk population	1 study
Hemodialysis patients	1 study

**Table 4:** The current markers analyzed over time.

Year	Biomarkers
2011	Homoarginine
2012	Serum 25-hydroxyvitamin D
2014	Thyroid-stimulating hormone (TSH)
	NT-proBNP Galectin 3 and ST2
2016	Soluble ST2
2017	Fasting plasma leptin
2018	9-cis-retinoic acid (9cRA)
	High-sensitivity troponin T, growth differentiation factor-15, NT-proBNP
2020	Total to high-density lipoprotein cholesterol ratio, high-sensitivity cardiac troponin I, NT-proBNP (N-terminal pro-B-type natriuretic peptide), or high-sensitivity C-reactive protein individually or in combination
2021	Albumin, uric acid, total bilirubin
	Plasma Omega-3 Fatty Acids
2023	MicroRNA-208b-3p MicroRNA-143-3p

## Data Availability

PubMed Database.
